# Enhanced versus standard fortification of pasteurized donor human milk for growth in very low birth weight infants: a randomized controlled trial

**DOI:** 10.3389/fnut.2025.1582519

**Published:** 2025-08-18

**Authors:** Chengsi Ong, Anng Anng Wong, Siew Tin Wong, Ying Zheng, Cynthia Pui Chan Pang, Pooja Agarwal Jayagobi, Joo Guan Yeo, Kee Thai Yeo, Mei Chien Chua

**Affiliations:** ^1^Department of Nutrition and Dietetics, KK Women's and Children's Hospital, Singapore, Singapore; ^2^KK Human Milk Bank, Singapore, Singapore; ^3^Division of Nursing, Lactation Services, Singapore, Singapore; ^4^Department of Neonatology, KK Women's and Children's Hospital, Singapore, Singapore; ^5^Duke-NUS Medical School, Singapore, Singapore; ^6^Division of Medicine, KK Women's and Children's Hospital, Singapore, Singapore; ^7^SingHealth Duke-NUS Academic Medical Centre, Translational Immunology Institute, Singapore, Singapore

**Keywords:** donor breast milk, growth, very low birth weight, preterm, fortification

## Abstract

**Introduction:**

Very-low-birthweight (VLBW) infants on pasteurized donor human milk (PDHM) have poorer growth compared to infants on fortified mother's milk, suggesting that standard fortification methods for PDHM are inadequate.

**Methods:**

We designed a randomized controlled trial to determine whether an enhanced method of fortification (EF) improved growth in VLBW infants compared to standard fortification (SF). VLBW infants admitted to our tertiary-level neonatal intensive care unit were randomized to receive a bovine powdered human milk fortifier (HMF) added to PDHM (SF), or specially selected high-fat PDHM (fat concentration ≥3.8 g/dL) with bovine powdered HMF and a liquid protein fortifier providing an additional 0.67 g/dL protein (EF). Primary outcome was impaired weight gain defined as weight z-score drop of ≥0.8 from birth at 37 weeks or hospital discharge, whichever earlier. Secondary outcomes included change in length and head circumference (HC) z-scores from birth, requirement for high calorie formula, and rates of bronchopulmonary dysplasia (BPD) and retinopathy of prematurity (ROP).

**Results:**

A total of 61 infants were randomized (31 SF, 30 EF). Impaired weight gain was not significantly different (SF 83.9% vs. EF 73.3%, p = 0.347), with similar declines in weight z-scores from birth in both groups SF −1.27 [interquartile range (IQR) −1.71, −0.87] vs. EF −1.13 (IQR −1.46, −0.78), *p* = 0.403. However, the EF group had a smaller decline in length and HC z-scores from birth to discharge compared to the SF group [Length z-score change: −0.92 (IQR −1.64, −0.48) vs. −1.64 (IQR −2.21, −0.89), *p* = 0.007; HC z-score change: −0.08 (IQR −0.74,0.58) vs. −0.86 (IQR −1.81, −0.21), *p* = 0.014]. The EF group also required less high calorie formula supplementation [0% (IQR 0-4.1) vs. 3.8% (IQR 0 −16.9), *p* = 0.032]. Rates of BPD and ROP were not significantly different between groups.

**Conclusion:**

Among VLBW infants, EF did not improve weight gain, but reduced declines in HC and linear growth compared to SF.

## 1 Introduction

Own mother's breastmilk is the ideal source of nutrition in preterm infants, as it contains bioactive factors and enzymes to promote growth and immunity in these babies (1). Among preterm infants without sufficient mother's own milk (MOM), pasteurized donor human milk (PDHM) is the preferred feed as it is protective against necrotizing enterocolitis (NEC), a potentially devastating gastrointestinal disease (2, 3). Even so, the use of PDHM has been associated with poorer growth among preterm, very low birth weight (VLBWs) infants compared to unpasteurized MOM (4, 5). One possible reason for this is the reduction in specific proteins, enzymes and growth promoting factors due to heat during pasteurization (6). Another possible reason is that PDHM is often obtained from mothers with term infants and is donated at a time when the child is older (7). As such, the macronutrient composition of PDHM, particularly protein, is often lower than in preterm mother's milk (8, 9), and insufficient to meet growth requirements of the preterm infant (10). Yet, experts opine that poor growth should not deter the use of PDHM (11), considering its protective effect against NEC.

Standard fortification of human milk for preterm infants involves the addition of human milk fortifier (HMF) in a standard manner regardless of the native human milk nutrient composition, usually according to manufacturer recommendations. Considering the nutrient differences between MOM and PDHM, there is a need for PDHM to be fortified differently from MOM in order to meet growth requirements of preterm infants. However, there are currently no specialized PDHM fortifiers or fortification recommendations.

Alternative methods of individualizing fortification of human milk—adjustable and targeted fortification—have been proposed to achieve higher nutrient delivery for growth in VLBW infants. Adjustable fortification involves increasing or decreasing fortification to keep the infant's blood urea nitrogen within an ideal target (12). Targeted fortification involves repeated macronutrient analysis of human milk such that specific amounts of modular macronutrient additives can be added to meet a target nutrient range (13). These two methods have limitations, namely frequent blood sampling and intensive labor, precluding their use in many units. Hence, we explored a third alternative method of PDHM fortification, termed “enhanced fortification”, that was less invasive and labor intensive, utilizing selection of higher nutrient PDHM from our milk bank with additional protein fortification. In this study, we aimed to determine whether our enhanced fortification method improved growth parameters in VLBW infants when compared to standard fortification.

## 2 Materials and methods

### 2.1 Study design and participants

This was an investigator-initiated, prospective, single-center, two-arm, parallel-group randomized controlled trial conducted between June 2021 and September 2023. This trial was registered at clinicaltrials.gov (NCT04640805) and we reported this trial following the CONSORT 2020 guidelines (Supplementary material) (14). Infants admitted to the tertiary-level neonatal intensive care unit (NICU) at KK Women's and Children's Hospital (KKH), Singapore within the first week of life were eligible for inclusion in the study. The initial inclusion criteria included infants with birth weight of ≤ 1,500 g achieving at least 40 ml/kg/day of feeds by day 7 of life, and requiring at least 25% of feeds as PDHM in the week before randomization. However, due to low eligibility, the inclusion criteria was revised to include VLBW infants achieving at least 40 ml/kg/day of feeds by day 14 of life, and requiring at least 25% of feeds as PDHM in the week before randomization. All infants were recruited after the change in inclusion criteria. Infants with diagnosed or suspected inborn errors of metabolism, congenital diseases or major malformations impacting growth (e.g., trisomy 21, neonatal encephalopathy and seizures, neonatal tumors, complex congenital heart disease, gastrointestinal disorders), and those with acute or chronic renal impairment, were excluded. All study procedures were carried out in accordance with the Helsinki Declaration, and ethics approval was obtained from the Singhealth Centralized Institutional Review Board (CIRB Ref: 2020/2493). Written informed consent was obtained from all legal guardians before study participation.

### 2.2 Randomization

Simple randomization was performed using an online random sequence generator by a study coordinator, and the allocation sequence was concealed in a sealed envelope. Participants were randomly assigned to receive either standard fortification of PDHM (SF) or enhanced fortification of PDHM (EF) in a 1:1 ratio. Twins or triplets were randomized to the same study group. Parents and clinicians (doctors, nurses, dietitians, therapists) directly involved in the participant's medical care were blinded to the study intervention; only nurses in charge of feed preparation and milk bank staff were aware of the intervention allocation.

### 2.3 Procedures

#### 2.3.1 Unit feeding practice

Feeds were escalated according to our NICU's enteral nutrition protocol (Supplementary Table S1). Briefly, all VLBW infants were supported on parenteral nutrition within 4–6 h of birth, and human milk feeds were initiated on day 1 of life, where possible. Feeds were graded up by 10–20 ml/kg/day per day over the subsequent days to reach a final volume of 160–180 ml/kg/day, as determined by the medical team. At a feed volume of 80 ml/kg/day, both PDHM and MOM were fortified using a commercial powdered bovine HMF (Similac HMF, Abbott Nutrition, Illinois, USA or PreNAN HMF, Nestle, Switzerland) to achieve 80kcal/dL (i.e., 1 sachet to every 25 ml of milk). This provided an additional 14 kcals and 1 g protein per 100 ml (Similac HMF) or 17 kcals and 1.44 g protein per 100 ml (PreNAN HMF). Where MOM was insufficient, PDHM was provided as the alternative choice of feed. All VLBW infants with insufficient MOM were eligible to receive PDHM until at least a post menstrual age (PMA) of 37 weeks.

PDHM was provided by the KKH Human Milk Bank, and was from mothers with infants below 1 year of age. At the milk bank, milk from a single donor was pooled and pasteurized using the Holder method (62.5°C for 30 mins) (15). After pasteurization, each batch of milk was analyzed for energy, fat, true protein and carbohydrate content using a mid-infrared human milk analyser (MIRIS, Uppsala, Sweden).

#### 2.3.2 Intervention

Infants with insufficient MOM in the SF group received PDHM as per standard of care, provided by the milk bank on a first-in first-out approach. The EF group received high-fat PDHM, defined as PDHM with a fat concentration of ≥3.8 g/dL. This cut-off for high-fat PDHM was chosen based on a historical median fat concentration of 3.8 g/dL at the milk bank.

In addition, the EF group received an additional 1 ml of an extensively hydrolysed liquid protein fortifier (Similac Liquid Protein Fortifier, Abbott Nutrition, Illinois, USA) to every 25 ml of PDHM, providing an additional 0.67 g protein per 100 ml. The amount of protein fortification was chosen to meet recommended protein concentrations for preterm infants by the European Society for Pediatric Gastroenterology Hepatology and Nutrition (ESPGHAN) (Table 1) (10). This amount of protein was also easy for the feed nurses to prepare, with 1 ml of liquid protein and 1 sachet of HMF added to every 25 ml of PDHM. No additional protein was provided in the SF group.

**Table 1:** Average macronutrient composition for standard (control) and enhanced fortification (intervention) groups.

**Values per 100 ml PDHM**	**Standard PDHM^a^**	**High-fat PDHM^a^**	**Standard fortification^b^**	**Enhanced fortification^b^**	**Recommended^c^**
Energy (kcals)	67.5 (66.2–68.6)	75.9 (74.1–77.5)	81.5 (80.2–82.6)	92.6 (90.8–94.1)	77–93
Protein (g)	0.78 (0.72–0.82)	0.82 (0.78–0.89)	1.78 (1.72–1.82)	2.49 (2.45–2.56)	2.3–2.7
Fat (g)	3.33 (3.16–3.45)	4.22 (4.11 −4.38)	3.69 (3.52–3.8)	4.58 (4.47–4.74)	3.2–5.4
Carbohydrate (g)	8.10 (8.01–8.15)	8.01 (7.93–8.07)	9.90 (9.80–9.95)	9.80 (9.73–9.87)	7.3–10.0
Protein: energy ratio	1.16 (1.11–1.21)	1.09 (1.02–1.16)	2.19 (2.14–2.23)	2.72 (2.65–2.78)	2.8–3.6

European Society for Paediatric Gastroenterology Hepatology and Nutrition; PDHM pasteurized donor human milk.

^**a**^Nutrient values for Standard and High-fat PDHM were measured using the MIRIS human milk analyzer.

^**b**^Standard fortification assumes the use of a powdered human milk fortifier (Similac HMF, Abbott Nutrition) while enhanced fortification involves use of a human milk fortifier and an additional protein supplement.

^**c**^Recommendations are according to European Society for Paediatric Gastroenterology Hepatology and Nutrition 2022 guidelines, assuming a conservative intake of 150 ml/kg/day.

The study interventions occurred until 37 weeks PMA or hospital discharge, whichever was earlier. During this period, participants in both groups were reviewed by dietitians weekly per unit protocol, and where growth was determined to be suboptimal, high-calorie preterm formula (Similac Special Care 30, Abbott Nutrition, Columbus, OH, USA) was used to replace up to half of human milk feeds as per dietitian recommendations. After 37 weeks PMA, infants in the EF group reverted to the standard of care, i.e., high-fat or standard-fat PDHM on a first-in first-out approach, and protein fortification was stopped.

### 2.4 Outcomes

Our primary outcome was impaired weight gain, defined as a weight z-score drop of 0.8 or more from birth on the 2013 Fenton charts, at 37 weeks PMA or hospital discharge, whichever is earlier (16, 17). For ease of interpretation, weight gain velocity was also calculated by averaging weekly weight gain (expressed as g/kg/day) over the same period. Secondary outcomes included change in length and HC z-scores from birth at 37 weeks PMA or hospital discharge, and the amount of high-calorie formula use. Incidence of Bronchopulmonary dysplasia (BPD) and retinopathy of prematurity (ROP) were also collected as secondary outcomes as they have been associated with growth and human milk feeding (18, 19). We originally planned for body composition to be measured using air displacement plethysmography; however, due to technical issues with our machine that were beyond repair, triceps skinfold thickness and mid-upper arm circumference measurements were used as an indicator of body composition instead (20, 21).

Weights were measured using a portable infant scale (SECA 727, SECA Corp, Germany), length was measured using an infant length board (SECA 727, SECA Corp, Germany) and head circumference was measured using a paper tape by trained bedside nurses. Triceps skinfold (TSF) thickness was measured using a Holtain caliper (Holtain, Crosswell, UK) and mid-upper arm circumference (MUAC) using a paper tape by a research coordinator. BPD was defined as the need for any oxygen support including nasal cannula, continuous positive airway pressure (CPAP) or mechanical ventilation at 36 weeks PMA (22), and the presence of ROP (stage 1 or above) was determined by independent ophthalmologists (23). Patient demographics, characteristics and actual volumes of MOM, PDHM and formula delivered to participants were collected from medical records. Estimated energy and protein intake from milk feeds were calculated for the duration of the study. For the purpose of estimating nutritional intake from feeds, we assumed an energy and protein concentration of 67 kcals and 1.2 g per 100 ml of MOM (24).

### 2.5 Statistical analysis

Based on a retrospective review of our unit data, baseline rates of impaired weight gain in VLBW infants requiring PDHM was found to be 60% vs. 25% in those who did not. To detect a reduction of impaired weight gain from 60% in our control group to 25% in our intervention group with a power of 80% and type 1 error of 5%, a sample size of 30 participants in each arm was required.

Statistical analysis was performed using IBM SPSS software v20 (SPSS Inc., Chicago, IL) with a two-tailed test and *p* < 0.05 considered to be statistically significant. Continuous variables were compared between groups using the Student's *t*-test and Mann-Whitney test for normal and skewed distributions respectively. Categorical variables were compared using the chi-square test or Fisher's exact test. Both intention-to-treat and per-protocol analyses were performed. A subgroup analysis was also performed in singleton pregnancies alone.

## 3 Results

**Figure 1:**
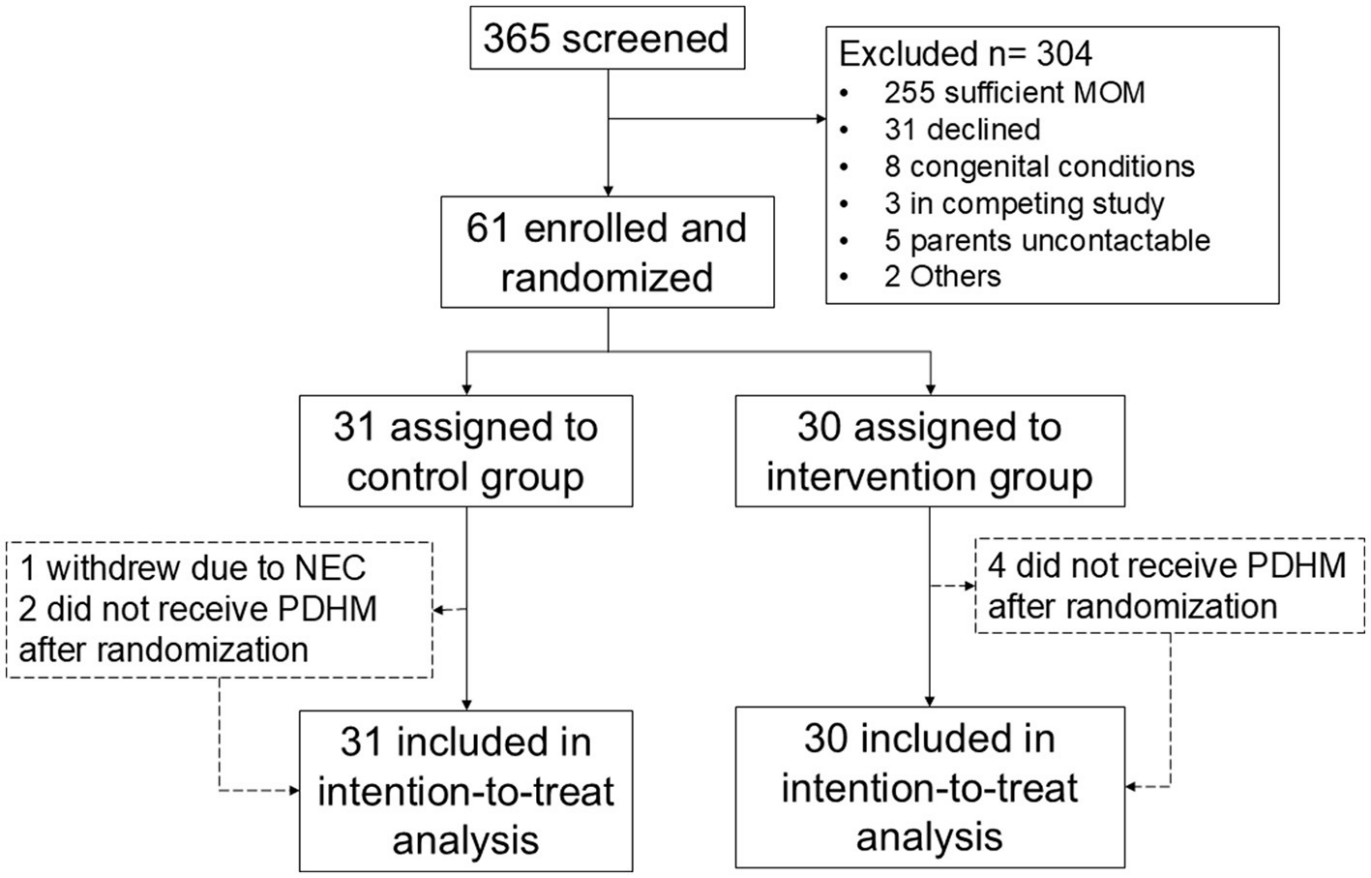
Flowchart of study participants.

A flow chart of subjects screened and recruited is shown in Figure 1. Between June 2021 and July 2023, a total of 61 infants were randomized, of which 31 were in the SF group and 30 in the EF group. Recruitment was temporarily stopped between 21st February 2022 and 20th September 2022 due to an international recall of Similac HMF, and lack of an alternative brand of HMF in our country. After September 2022, PreNan HMF was used, and similar numbers in both groups received PreNAN HMF (22/30, 71.0% in the SF group vs. 22/31, 73.3% in the EF group, *p* = 0.837). All participants completed the study procedures by September 2023. Baseline characteristics of participants in both groups are comparable as shown in Table 2, except for a higher number of multiple pregnancies in the SF group. Three participants in the SF group did not complete the study, while four in the EF group did not complete the study.

**Table 2:** Baseline characteristics of intention-to-treat population.

**Variables**	**Control: standard fortification (*n* = 31)**	**Intervention: enhanced fortification (*n* = 30)**
Cesarean delivery, *n* (%)	28 (90.3)	23 (76.7)
Birth gestation, weeks	28.9 (28.1–31.0)	29.0 (28.5–31.3)
Male sex, *n* (%)	16 (51.6)	15 (50.0)
**Ethnicity**		
Chinese	14 (45.2)	21 (70.0)
Malay	5 (16.1)	4 (13.3)
Indian	7 (22.6)	3 (10.0)
Others	5 (16.1)	2 (6.7)
5-min Apgar score	9 (8–9)	9 (8–9)
Birth weight, g	1,175 (990–1,305)	1,320 (1,062–1,433)
Birth length, cm	38.0 (35.0–39.5)	37.5 (35.0–39.0)
Birth head circumference, cm	26.0 (25.0–27.5)	26.5 (25.0–27.5)
Small for gestational age, *n* (%)	7 (22.6)	3 (10.0)
Multiple pregnancy, *n* (%)^*^	12 (38.7)	3 (10.0)
Duration of invasive mechanical ventilation, hours	5.0 (0–35.0)	0 (0–10.0)
Day of life feeds started	1.0 (0–1.0)	0 (0–1.0)
Days of parenteral nutrition^*^	9.0 (6.0–11.0)	6.0 (4.0–9.3)
**Human milk fortifier used**, ***n*** **(%)**		
Similac	9 (29.0)	8 (26.7)
PreNAN	22 (71.0)	22 (73.3)
Necrotizing enterocolitis, *n* (%)	1 (3.2)	0 (0)
Percentage of feeds as PDHM	41.6 (13.5–75.5)	58.8 (18.0–76.7)
Percentage of feeds as MOM	40.3 (14.0–75.5)	36.9 (14.3–81.7)

^*^Baseline characteristics were similar between groups (*p* ≥ 0.05), except for higher number of multiple pregnancies (*p* = 0.031), and more days of parenteral nutrition (*p* = 0.006) in the control group compared to intervention group.

Values are expressed as median (interquartile range) unless otherwise specified. MOM, mother's own milk; PDHM, pasteurized donor human milk.

Macronutrient analysis of PDHM provided to the SF and EF group are shown in Table 1. PDHM provided to the EF group was significantly higher in energy, fat and protein compared to PDHM provided to the SF group. PDHM proportions of total feeds were similar between groups [SF: 41.6% (13.5–75.5%) vs. EF: 58.8% (18.0–76.7), *p* = 0.471]. Likewise, MOM constituted a similar proportion of total feeds in both groups [SF: 40.3% (IQR 14.0–75.5) vs. EF: 36.9% (IQR 14.3–81.7), *p* = 0.997]. There was one case of NEC in the SF group, and none reported in the EF group.

Intention-to-treat analysis demonstrated that rates of impaired weight gain (83.9% vs. 73.3%, *p* = 0.347) and weight z-score change from birth [−1.27 (IQR −1.71 to −0.87) vs. −1.13 (IQR −1.46 to −0.78), *p* = 0.403] were not significantly different between SF and EF groups (Table 3). However, the EF group experienced smaller declines in length z–scores and HC z–scores from birth compared to the SF group [length: −0.92 (IQR −1.64 to −0.48) vs. −1.64 (IQR −2.21 to −0.89), p = 0.007; HC: −0.08 (IQR −0.74–0.58) vs. −0.86 (IQR −1.81 to −0.21), *p* = 0.014]. There was also less use of high–calorie formula as a percentage of total feed volume in the EF compared to the SF group [0% (IQR 0–4.1) vs. 3.8% (IQR 0–16.9), *p* = 0.032].

**Table 3:** Outcomes based on intention-to-treat analysis.

**Outcomes**	**Control: standard fortification (*n* = 31)**	**Intervention: enhanced fortification (*n* = 30)**	***P*-value**
Impaired weight gain, *n* (%)	26 (83.9)	22 (73.3)	0.347
Weight gain velocity, g/kg/day	12.5 (9.7–14.5)	11.5 (8.7–13.6)	0.306
Change in weight z-score from birth	−1.27 (−1.71 to −0.87)	−1.13 (−1.46 to −0.78)	0.403
Change in length z-score from birth	−1.64 (−2.21 to −0.89)	−0.92 (−1.64 to −0.48)	0.007
Change in HC z-score from birth	−0.86 (−1.81 to −0.21)	−0.08 (−0.74–0.58)	0.014
%High-calorie formula use	3.8 (0–16.9)	0 (0–4.1)	0.032
Hospital LOS, days	63 (48–86)	57 (38–71)	0.085
BPD, *n* (%)	13 (41.9)	6 (20.0)	0.064
ROP, *n* (%)	10 (32.3)	7 (23.3)	0.437
Triceps skinfold thickness, mm	4.6 (3.6–5.5)	4.3 (3.5–5.2)	0.635
Mid upper arm circumference, cm	8.6 (8.0–9.9)	8.5 (8.0–9.5)	0.949

BPD, bronchopulmonary dysplasia; HC, head circumference; LOS, length of stay; ROP, retinopathy of prematurity. Continuous values are expressed as median (interquartile range, IQR) and categorical variables are expressed as counts (percentages, %).

The per-protocol analysis included 28/31 from the SF group and 26/30 from the EF group (Supplementary Table S2). Similar results were observed in the per-protocol analysis as compared to the intention-to-treat analysis, where the EF group had smaller declines in length and head circumference z-scores from birth, and lower high-calorie formula use compared to the SF group. In the subgroup analysis of singleton pregnancies, the EF group similarly had smaller declines in length and head circumference z-scores from birth compared to the SF group, but the amount of high-calorie formula use was not significantly different between groups (Supplementary Table S3).

## 4 Discussion

Despite the common use of PDHM in preterm infants, there has been limited guidance on the fortification of PDHM to optimize growth among VLBW infants. In our RCT, we explored an enhanced method of PDHM fortification by selecting PDHM with higher fat concentration, and addition of protein, in order to optimize growth. While our enhanced fortification method did not significantly improve weight z-scores at discharge, it resulted in smaller declines in length and HC z-scores at discharge, suggesting that our fortification method was able to promote specific growth parameters in VLBW infants.

Two main methods of individualizing human milk fortification in VLBW infants include adjustable and targeted fortification. Unlike standard fortification which assumes a standard macronutrient profile of milk, individualized fortification accounts for the variability in human milk. Individualized fortification has been shown to improve weight, length and head circumference growth as well as body composition in VLBW infants (25). The effect on other neonatal comorbidities including bronchopulmonary dysplasia and retinopathy of prematurity have also been studied, albeit without significant results (26, 27). However, a main limitation of these methods is the need for frequent measurements and adjustments to the feed regime, requiring a significant amount of manpower. We utilized a hybrid of standard and individualized fortification, which took into account the variability in human milk composition used at our unit. We selected PDHM based on fat and not protein concentrations as we found a wider variability in fat concentrations. In addition, protein concentrations were generally low, indicating that additional protein supplementation were still necessary to meet recommended intakes. The advantage of our fortification method is that it is less manpower-intensive compared to traditional standard fortification methods; additional manpower is only required for nutrient analysis of PDHM, which can be achieved relatively quickly using a bedside milk analyser.

The primary goal of our fortification strategy was to reduce impaired weight gain, as weight gain is associated with better organ development, reduced respiratory dependence and better neurodevelopmental outcomes in VLBW infants (28, 29). Aside from weight gain, length and HC are important growth parameters as they have been shown to reflect lean body mass deposition and long-term neurodevelopment (30, 31). In our study, we observed smaller declines in length and HC z-scores from birth in the EF group compared to SF in both intention-to-treat and per-protocol analyses, suggesting that our fortification method has a stronger effect on length and HC compared to weight. It is unclear whether this effect was attributable to an increase in protein as a percentage of total energy provision, or an overall increase in energy intake from fat in the intervention group. A systematic review which evaluated the effect of higher protein and similar calories on growth in preterm infants found that higher protein was shown to have a more consistent effect on weight than length and head circumference when calories were kept the same (32). Interestingly, an observational study of preterm infants that analyzed macronutrient compositions of MOM and PDHM found that higher fat intake from breastmilk was associated with better head growth in the first 2 months of life (33), suggesting the potential advantage of a higher proportion of fat as a source of energy. Indeed, higher fat intakes have been associated with greater brain volume and better neurodevelopment in preterm infants (34). This is consistent with our findings of maintenance in HC z-scores from birth to discharge in the EF but not SF group (35–37). Isolation and use of human milk fat as an additive for PDHM may be a useful fortification method for donor milk banks in future studies. However, it is important to note that despite a smaller decline, reductions in length were still observed in the EF group, suggesting that our EF strategy was still insufficient to promote adequate growth in VLBW infants.

The ESPGHAN recommends an intake of 3.5 to 4.5 g/kg/day to promote growth in VLBW infants, with extremely low birth weight infants likely requiring a higher intake than VLBW infants (10, 38). PDHM concentrations in our cohort were low and were unable to meet recommended protein intakes without additional supplementation, as observed in the SF group, which could have explained the poorer linear and HC growth. There is thus a need for further study on protein fortification of PDHM to inform recommendations on supplementation strategies. In an observational pre-post cohort study, Fu et al. compared standard fortified PDHM against fortified PDHM with an additional 1 g/dL of protein in VLBW infants for the first 30 days of life (39). The authors did not observe better weight gain in infants provided additional protein, although this may have been because additional formula was used to increase the concentration of PDHM to promote growth. Similarly, an RCT comparing a standard with a higher protein HMF (1 g vs. 1.8 g/dL) did not improve weight, length or HC growth (40). Our fortification strategy increased the protein content by 0.67 g per 100 ml of PDHM, which was within the ESPGHAN recommended protein intake at an assumed feed volume of 150 ml/kg/day, but below levels that were previously studied (10). In an RCT exploring additional protein fortification, a protein intake of 4.6 g/kg/day was found to improve weight gain velocity compared to an intake of 3.7 g/kg/day (41). To achieve a protein intake of 4.5 g/kg/day in our EF group, an additional fortification of 0.6 g of protein per 100 ml of PDHM would have been required, assuming feed volumes of 150 ml/kg/day.

We recognize that selecting high-fat PDHM may not be possible for units that rely on external milk banks for PDHM provision and are unable to control the milk selection process. Additionally, a limitation of our fortification method is that only a proportion of the PDHM is utilized, adequate measures are needed to ensure the remaining the PDHM at our milk bank is not wasted. While we were able to use the PDHM with standard fat content for older preterm infants and term infants, care needs to be taken to prevent wastage of PDHM that is not used. Other pooling and distribution methods to optimize PDHM nutrient content and subsequently growth in preterm infants can be utilized, depending on the model of the milk bank. For example, the Human Milk Bank Association of North America recommends pooling from multiple-donors, which differs from the Australian milk bank guidelines which recommend pooling from a single donor (42, 43). Multiple-donor pooling is likely to result in less inter-batch variability, and targeted donor pooling (combining specific batches to achieve the desired nutrient composition) has been recommended as a way to optimize the nutrient content of PDHM (44). However, it is likely that additional supplementation, particularly that of protein, will still be required after pooling (7). In another innovative study, preterm PDHM was provided to VLBW infants in the first 3 weeks of life, which improved their protein intake, body weight and head circumference compared to those who were provided with term PDHM (45). This suggests that the selection of milk by lactation age may also be a feasible way to improve growth in VLBW infants requiring PDHM.

There are several limitations of our study. First, we only looked at short-term growth outcomes at hospital discharge. Longer-term consequences of the provision of breastmilk with high-fat in the early days are unclear, although our fat concentration was still within physiological ranges for breastmilk, and similar to preterm MOM. In addition, we did not observe higher triceps skinfold thickness values in the intervention group, suggesting that body composition was not significantly impacted, at least in the short term. Second, we did not measure macronutrient composition of MOM, and was thus unable to determine whether actual energy and protein intakes were higher in the EF group compared to the SF group. However, based on the higher measured PDHM nutrient concentrations in the EF compared to the SF group (Table 1), and a comparable proportion of feeds as PDHM in both groups, total energy and protein intakes were likely higher in the EF compared to the SF group. Third, we used TSF and MUAC as proxy measurements of body composition, which can have wide inter-operator dependence and may not reflect whole body fat percentage (46). However, it is a relatively low-cost tool that can easily be adopted by studies, until more accurate and accessible methods of body composition measurement for preterm infants can be established.

## 5 Conclusion

Enhanced fortification of PDHM, by selecting PDHM with fat ≥3.8 g/dL and additional protein fortification of 0.67 g/dL, resulted in better length and HC growth but not weight gain in VLBW infants compared to standard fortification. This method of fortification, which is not as labor-intensive as individualized fortification, will still need further optimization to be a suitable strategy to optimize growth in VLBW infants requiring PDHM.

## Data Availability

The raw data supporting the conclusions of this article will be made available by the authors, without undue reservation.

## References

[1] Eidelman AI Schanler RJ Johnston M Landers S Noble L Szucs K. Breastfeeding and the Use of Human Milk. Pediatrics. (2012) 129:e827–e41. 10.1542/peds.2011-3552 22371471

[2] Miller J Tonkin E Damarell RA McPhee AJ Suganuma M Suganuma H. A systematic review and meta-analysis of human milk feeding and morbidity in very low birth weight infants. Nutrients. (2018) 10:707. 10.3390/nu10060707 29857555 PMC6024377

[3] de Halleux V Pieltain C Senterre T Studzinski F Kessen C Rigo V. Growth benefits of own mother's milk in preterm infants fed daily individualized fortified human milk. Nutrients. (2019) 11:772. 10.3390/nu11040772 30987136 PMC6521225

[4] Madore LS Bora S Erdei C Jumani T Dengos AR Sen S. Effects of donor breastmilk feeding on growth and early neurodevelopmental outcomes in preterm infants: an observational study. Clin Ther. (2017) 39:1210–20. 10.1016/j.clinthera.2017.05.341 28576299

[5] Brownell EA Matson AP Smith KC Moore JE Esposito PA Lussier MM. Dose-response relationship between donor human milk, mother's own milk, preterm formula, and neonatal growth outcomes. J Pediatr Gastroenterol Nutr. (2018) 67:90–6. 10.1097/MPG.0000000000001959 29543698

[6] Peila C Moro GE Bertino E Cavallarin L Giribaldi M Giuliani F. The effect of holder pasteurization on nutrients and biologically-active components in donor human milk: a review. Nutrients. (2016) 8:477. 10.3390/nu8080477 27490567 PMC4997390

[7] Fu TT Schroder PE Poindexter BB. Macronutrient analysis of target-pooled donor breast milk and corresponding growth in very low birth weight infants. Nutrients. (2019) 11:1884. 10.3390/nu11081884 31412627 PMC6722642

[8] Gates A Hair AB Salas AA Thompson AB Stansfield BK. Nutrient composition of donor human milk and comparisons to preterm human milk. J Nutr. (2023) 153:2622–30. 10.1016/j.tjnut.2023.07.012 37517552

[9] Perrin MT Belfort MB Hagadorn JI McGrath JM Taylor SN Tosi LM. The nutritional composition and energy content of donor human milk: a systematic review. Adv Nutr. (2020) 11:960–70. 10.1093/advances/nmaa014 32119744 PMC7360450

[10] Embleton ND Jennifer Moltu S Lapillonne A van den Akker CHP Carnielli V Fusch C. Enteral nutrition in preterm infants (2022): a position paper from the espghan committee on nutrition and invited experts. J Pediatr Gastroenterol Nutr. (2023) 76:248–68. 10.1097/MPG.0000000000003642 36705703

[11] Picaud JC. Review highlights the importance of donor human milk being available for very low birth weight infants. Acta Paediatr. (2022) 111:1127–33. 10.1111/apa.16296 35170785 PMC9314126

[12] Alan S Atasay B Cakir U Yildiz D Kilic A Kahvecioglu D. An intention to achieve better postnatal in-hospital-growth for preterm infants: adjustable protein fortification of human milk. Early Hum Dev. (2013) 89:1017–23. 10.1016/j.earlhumdev.2013.08.015 24035039

[13] Arslanoglu S Boquien CY King C Lamireau D Tonetto P Barnett D. Fortification of human milk for preterm infants: update and recommendations of the european milk bank association (EMBA) working group on human milk fortification. Front Pediatr. (2019) 7:76. 10.3389/fped.2019.00076 30968003 PMC6439523

[14] Moher D Hopewell S Schulz KF Montori V Gøtzsche PC Devereaux PJ. CONSORT 2010 explanation and elaboration: updated guidelines for reporting parallel group randomised trials. BMJ. (2010) 340:c869. 10.1136/bmj.c869 20332511 PMC2844943

[15] Landers S Updegrove K. Bacteriological screening of donor human milk before and after Holder pasteurization. Breastfeed Med. (2010) 5:117–21. 10.1089/bfm.2009.0032 20509779

[16] Fenton TR Kim JH. A systematic review and meta-analysis to revise the Fenton growth chart for preterm infants. BMC Pediatr. (2013) 13:59. 10.1186/1471-2431-13-59 23601190 PMC3637477

[17] Goldberg DL Becker PJ Brigham K Carlson S Fleck L Gollins L. Identifying malnutrition in preterm and neonatal populations: recommended indicators. J Acad Nutr Diet. (2018) 118:1571–2. 10.1016/j.jand.2017.10.006 29398569

[18] Goyal K Arya S Guliani B. Effect of breast milk feeding on retinopathy of prematurity in neonates less than 1800 grams: a cohort. Journal of Neonatology. (2024) 38:52–9. 10.1177/09732179231220208

[19] Rocha G Guimarães H Pereira-da-Silva L. The role of nutrition in the prevention and management of bronchopulmonary dysplasia: a literature review and clinical approach. Int J Environm Res Public Health. (2021) 18:6245. 10.3390/ijerph18126245 34207732 PMC8296089

[20] Schmelzle HR Fusch C. Body fat in neonates and young infants: validation of skinfold thickness versus dual-energy X-ray absorptiometry1,2,3. Am J Clin Nutr. (2002) 76:1096–100. 10.1093/ajcn/76.5.1096 12399284

[21] Daly-Wolfe KM Jordan KC Slater H Beachy JC Moyer-Mileur LJ. Mid-arm circumference is a reliable method to estimate adiposity in preterm and term infants. Pediatr Res. (2015) 78:336–41. 10.1038/pr.2015.103 26020147

[22] Higgins RD Jobe AH Koso-Thomas M Bancalari E Viscardi RM Hartert TV. Bronchopulmonary dysplasia: executive summary of a workshop. J Pediatr. (2018) 197:300–8. 10.1016/j.jpeds.2018.01.043 29551318 PMC5970962

[23] Chiang MF Quinn GE Fielder AR Ostmo SR Paul Chan RV Berrocal A. International classification of retinopathy of prematurity, third edition. Ophthalmology. (2021) 128:e51–68. 10.1016/j.ophtha.2021.05.031 34247850 PMC10979521

[24] Nommsen LA Lovelady CA Heinig MJ Lönnerdal B Dewey KG. Determinants of energy, protein, lipid, and lactose concentrations in human milk during the first 12 mo of lactation: the DARLING Study. Am J Clin Nutr. (1991) 53:457–65. 10.1093/ajcn/53.2.457 1989413

[25] Fabrizio V Trzaski JM Brownell EA Esposito P Lainwala S Lussier MM. Individualized versus standard diet fortification for growth and development in preterm infants receiving human milk. Cochrane Database Syst Rev. (2020) 11:CD013465. 10.1002/14651858.CD013465.pub2 33226632 PMC8094236

[26] Kadioglu Simşek G Alyamaç Dizdar E Arayici S Canpolat FE Sari FN Uraş N. Comparison of the effect of three different fortification methods on growth of very low birth weight infants. Breastfeed Med. (2019) 14:63–8. 10.1089/bfm.2018.0093 30484683

[27] Sanchez-Holgado M Saenz de. Pipaon M, Jimenez MC, Crespo Sanchez G, Molero-Luis M, Montes MT, et al. Adjusted versus targeted fortification in extremely low birth weight preterm infants: fortin study—a randomized clinical trial. Nutrients. (2024) 16:2904. 10.3390/nu16172904 39275220 PMC11397412

[28] Williams E Dassios T Arnold K Hickey A Greenough A. Prolonged ventilation and postnatal growth of preterm infants. J Perinat Med. (2019) 48:82–6. 10.1515/jpm-2019-0278 31714891

[29] Ong KK Kennedy K Castañeda-Gutiérrez E Forsyth S Godfrey KM Koletzko B. Postnatal growth in preterm infants and later health outcomes: a systematic review. Acta Paediatr. (2015) 104:974–86. 10.1111/apa.13128 26179961 PMC5054880

[30] Raghuram K Yang J Church PT Cieslak Z Synnes A Mukerji A. Head growth trajectory and neurodevelopmental outcomes in preterm neonates. Pediatrics. (2017) 140:0216. 10.1542/peds.2017-0216 28759409

[31] Ramel SE Demerath EW Gray HL Younge N Boys C Georgieff MK. The relationship of poor linear growth velocity with neonatal illness and two-year neurodevelopment in preterm infants. Neonatology. (2012) 102:19–24. 10.1159/000336127 22441508

[32] Tonkin EL Collins CT Miller J. Protein intake and growth in preterm infants: a systematic review. Glob Pediatr Health. (2014) 1:2333794x14554698. 10.1177/2333794X14554698 27335914 PMC4804669

[33] Stoltz Sjöström E Öhlund I Ahlsson F Engström E Fellman V Hellström A. Nutrient intakes independently affect growth in extremely preterm infants: results from a population-based study. Acta Paediatr. (2013) 102:1067–74. 10.1111/apa.12359 23855971

[34] Coviello C Keunen K Kersbergen KJ Groenendaal F Leemans A Peels B. Effects of early nutrition and growth on brain volumes, white matter microstructure, and neurodevelopmental outcome in preterm newborns. Pediatr Res. (2018) 83:102–10. 10.1038/pr.2017.227 28915232

[35] Rochow N Raja P Liu K Fenton T Landau-Crangle E Göttler S. Physiological adjustment to postnatal growth trajectories in healthy preterm infants. Pediatr Res. (2016) 79:870–9. 10.1038/pr.2016.15 26859363

[36] Mayrink MLS Villela LD Méio M Soares FVM de Abranches AD Nehab SRG. The trajectory of head circumference and neurodevelopment in very preterm newborns during the first two years of life: a cohort study. J Pediatr (Rio J). (2024) 100:483–90. 10.1016/j.jped.2024.04.005 38806152 PMC11361857

[37] Cho H Kim E-K Song IG Heo JS Shin SH Kim H-S. Head growth during neonatal intensive care unit stay is related to the neurodevelopmental outcomes of preterm small for gestational age infants. Pediat Neonatol. (2021) 62:606–11. 10.1016/j.pedneo.2021.05.023 34266785

[38] Agostoni C Buonocore G Carnielli VP De Curtis M Darmaun D Decsi T. Enteral nutrient supply for preterm infants: commentary from the European Society of Paediatric Gastroenterology, Hepatology and Nutrition Committee on Nutrition. J Pediatr Gastroenterol Nutr. (2010) 50:85–91. 10.1097/MPG.0b013e3181adaee0 19881390

[39] Fu TT Kaplan HC Fields T Folger AT Gordon K Poindexter BB. Protein enrichment of donor breast milk and impact on growth in very low birth weight infants. Nutrients. (2021) 13:2869. 10.3390/nu13082869 34445027 PMC8401419

[40] Reid J Makrides M McPhee AJ Stark MJ Miller J Collins CT. The effect of increasing the protein content of human milk fortifier to 1.8 g/100 ml on growth in preterm infants: a randomised controlled trial. Nutrients. (2018) 10:634. 10.3390/nu10050634 29772833 PMC5986513

[41] Hemmati F Ghassemzadeh M. The effect of oral protein supplementation on the growth of very low birth weight preterm infants admitted to the neonatal intensive care unit: a randomized clinical trial. J Mother Child. (2023) 27:21–9. 10.34763/jmotherandchild.20232701.d-22-00072 37368944 PMC10298488

[42] Updegrove K Baker A Festival J Ginsberg H Hackney R Jones F. Standards for donor human milk banking: an overview. In: Human Milk Banking Association of North America (HMBANA). Fort Worth, TX: Human Milk Banking Association of North America (HMBANA) (2024).

[43] Care AGDoHaA. Operational guidelines for milk banks in Australia and New Zealand. Melbourne: Australian Government Department of Healthy, Disability and Ageing (2022).

[44] Tabasso C Piemontese P Pesenti N Perrone M Menis C Liotto N. Pooling strategies to modify macronutrient content of pasteurized donor human milk. Breastfeed Med. (2023) 18:370–6. 10.1089/bfm.2023.0043 37098175

[45] Gialeli G Kapetanaki A Panagopoulou O Vourna P Michos A Kanaka-Gantenbein C. Supplementation of mother's own milk with preterm donor human milk: impact on protein intake and growth in very low birth weight infants-a randomized controlled study. Nutrients. (2023) 15:566. 10.3390/nu15030566 36771273 PMC9919101

[46] Yumani DFJ de Jongh D Ket JCF Lafeber HN van Weissenbruch MM. Body composition in preterm infants: a systematic review on measurement methods. Pediatr Res. (2023) 93:1120–40. 10.1038/s41390-022-02262-x 35995939

