# Supplementary material for: Enhanced versus standard fortification of pasteurized donor human milk for growth in very low birth weight infants: a randomized controlled trial
Source: Front Nutr. 2025 Aug 18;12:1582519. doi: 10.3389/fnut.2025.1582519 (PMC12400863; doi:10.3389/fnut.2025.1582519)
Supplement: Supplementary file 1 [file Table_1.docx]

Supplementary Material

# Supplementary Table S1. Enteral nutrition escalation protocol

| **Birth Weight** | **Enteral nutrition volumes (ml/kg/day)*** | | | | | | **Remainder of feeds** |
| --- | --- | --- | --- | --- | --- | --- | --- |
|  | **Day 1** | **Day 2** | **Day 3** | **Day 4** | **Day 5** | **Day 6** |  |
| <1000 grams | Oral care or 10 | 20 | 40 | 60 | 80 | 100 | 20ml/kg/day advancement |
| 1000-1500 grams | 20 | 40 | 60 | 90 | 120 | 150 | 30ml/kg/day advancement |

# *Feeds are escalated as per protocol unless feed intolerance present

# Supplementary Table S2. Outcomes based on per-protocol analysis

| **Outcomes** | **Control: Standard fortification (n=28)** | **Intervention: Enhance fortification (n=26)** | **P-value** |
| --- | --- | --- | --- |
| Impaired weight gain, n (%) | 24 (85.7) | 20 (76.9) | 0.494 |
| Weight gain velocity, g/kg/day | 12.6 (9.7 – 14.4) | 11.3 (8.7 – 13.6) | 0.275 |
| Change in Weight z-score from birth | -1.28 (-1.69 – -0.90) | -1.22 (-1.55 – -0.79) | 0.591 |
| Change in Length z-score from birth | -1.68 (-2.21 – -0.96) | -1.00 (-1.67 – 0.61) | 0.013 |
| Change in Head Circumference z-score from birth | -0.86 (-1.77 - -0.23) | -0.08 (-0.74 – 0.48) | 0.021 |
| %High-calorie formula use | 5.42 (0 – 16.7) | 0 (0 – 4.1) | 0.040 |
| Hospital length of stay, days | 62.0 (45.0 – 81.0) | 56.5 (37.8 – 71.0) | 0.197 |
| Bronchopulmonary dysplasia, n (%) | 10 (35.7) | 4 (15.4) | 0.089 |
| Retinopathy of Prematurity, n (%) | 9 (32.1) | 6 (23.1) | 0.457 |
| Triceps skinfold thickness, mm | 4.35 (3.48 – 5.32) | 4.20 (3.45 – 5.08) | 0.595 |
| Mid upper arm circumference, cm | 8.50 (7.88 – 9.53) | 8.50 (7.88 – 9.53) | 0.985 |

# Values are expressed as median (interquartile range) unless otherwise specified

# Supplementary table S3. Subgroup intention-to-treat analysis for singleton pregnancies

| **Outcomes** | **Control: Standard fortification (n=19)** | **Intervention: Enhanced fortification (n=27)** | **P-value** |
| --- | --- | --- | --- |
| Impaired weight gain, n (%) | 17 (94.4) | 19 (70.4) | 0.064 |
| Weight gain velocity, g/kg/day | 12.1 (9.7 – 13.4) | 11.6 (9.3 – 13.9) | 0.729 |
| Change in Weight z-score from birth | -1.30 (-1.74 – -1.13) | -1.06 (-1.37 – -0.76) | 0.073 |
| Change in Length z-score from birth | -1.69 (-2.04 – -1.10) | -0.76 (-1.59 – -0.37) | 0.004 |
| Change in Head Circumference z-score from birth | -0.92 (-1.81 – -0.25) | -0.07 (-0.72 – 0.56) | 0.008 |
| %High-calorie formula use | 0 (0 – 11.1) | 0 (0 – 3.57) | 0.241 |
| Hospital length of stay, days | 61 (44 – 69) | 56 (38 – 71) | 0.304 |
| Bronchopulmonary dysplasia, n (%) | 6 (31.6) | 6 (22.2) | 0.513 |
| Retinopathy of Prematurity, n (%) | 5 (26.3) | 5 (18.5) | 0.719 |
| Triceps skinfold thickness, mm | 4.1 (3.3 – 5.3) | 4.3 (3.6 – 5.3) | 0.515 |
| Mid upper arm circumference, cm | 8.5 (7.4 – 9.3) | 8.8 (8 – 9.6) | 0.213 |

# Values are expressed as median (interquartile range) unless otherwise specified
